# Flatness and boundness of photonic drumhead surface state in a metallic lattice

**DOI:** 10.1038/s41598-021-88004-1

**Published:** 2021-04-22

**Authors:** Yu Wang, Xiaoxi Zhou, Shanshan Li, Wenya Zhang, Chuandeng Hu, Weixin Lu, Bo Hou

**Affiliations:** 1 School of Physical Science and Technology & Collaborative Innovation Center of Suzhou Nano Science and Technology, Soochow University, Suzhou, 215006 China; 2 Shenzhen Fantwave Tech. Co., Ltd, Shenzhen, 518110 China; 3 Wenzheng College of Soochow University, Suzhou, 215104 China; 4 Key Laboratory of Modern Optical Technologies of Ministry of Education & Key Lab of Advanced Optical Manufacturing Technologies of Jiangsu Province, Suzhou, 215006 China

**Keywords:** Metamaterials, Photonic crystals

## Abstract

Nodal chain (NC) semi-metals have the degeneracy of interlacing rings in their band structure in momentum space. With the projection of degenerate rings towards crystal boundaries, there is a special type of surface dispersion appearing at surface Brillouin zone and termed drumhead surface state (DSS). Previously, experimental investigations on photonic NC and DSS have been done on metallic photonic crystals at microwave frequencies. However, far-field detection of DSS and its coupling to radiative modes in free space have not been studied. In the work, we analyze the photonic DSS in a metallic lattice by angle-resolved far-field reflection measurement and numerical simulation at terahertz (THz) frequencies, and reveal its flatness and boundness in band structure, even in the radiation continuum. Particularly, the DSS band can be tuned being from negatively dispersive via flat to positively dispersive by a single surface parameter, and the DSS at Γ point in surface Brillouin zone is in fact a symmetry-protected bound state in the continuum. Our results might have some potential applications towards THz photonics.

## Introduction

Band theory is a cornerstone theory in solid state physics. For example, it reveals the physical mechanism classifying metals, semiconductors, and insulators in terms of an important concept, energy gap^1^. Recently, an intense research effort is contributed to energy degeneracy in band structures, because new physics and new applications are anticipated arising from peculiar band degeneracies, e.g. Dirac points and Weyl points^2,3^. Being parallel to electronic systems and de Broglie wave in solids, photonic crystals (PhCs) and metamaterials provide an arena of extending the band theory to electromagnetic (EM) wave, including band gap and nodal degeneracy^4–13^. Although being often an analogue to electronic systems, the relevant research has led to novel ideas and technologies of light manipulation^14–18^, relying on almost at-will designing flexibility in photonic crystals and metamaterials.

Among various band degeneracies, nodal chain (NC) is a configuration where two bands cross linearly in momentum space and the crossing points consist of a collection of interlacing rings^19,20^. Such degeneracy is challenging to be observed in electronic systems, but can be readily engineered and measured in an ordinary photonic structure, which is a three-dimensional (3D) metal-wire mesh with simple cubic lattice and exhibits the NC degeneracy in the band structure at microwave frequencies^21^. In addition to the NC in bulk band, the drumhead surface state (DSS), resultant from the projection of the NC towards the crystal boundary, was also found through near-field imaging on perfect-electric-conductor-enclosed (PEC-enclosed) surface of the metallic mesh^21^.

In this article, we have fabricated such a metallic lattice PhC in terahertz (THz) frequencies via 3D printing technology and subsequent chemical plating process, and have implemented angle-resolved far-field reflection measurement by the THz time-domain spectroscopy. Particularly different from the near-field approach to the PEC-enclosed surface in Ref.^21^, we have found the DSS persists to the far-field reflection where the crystal surface is exposed to radiative modes in free space, and have observed its flatness and boundness in the radiation continuum. Interestingly, the flatness of the DSS can be tuned through the extruding length of the sectioned metallic rod on the surface, and the DSS at Γ point in surface Brillouin zone is in fact a bound state in the continuum (BIC)^22–28^. Our results not only establish a connection between photonic topological surface states and BIC, but also explore an efficient technical route to fabricate and apply THz photonic structures.

## Model and experiment

**Figure 1:**
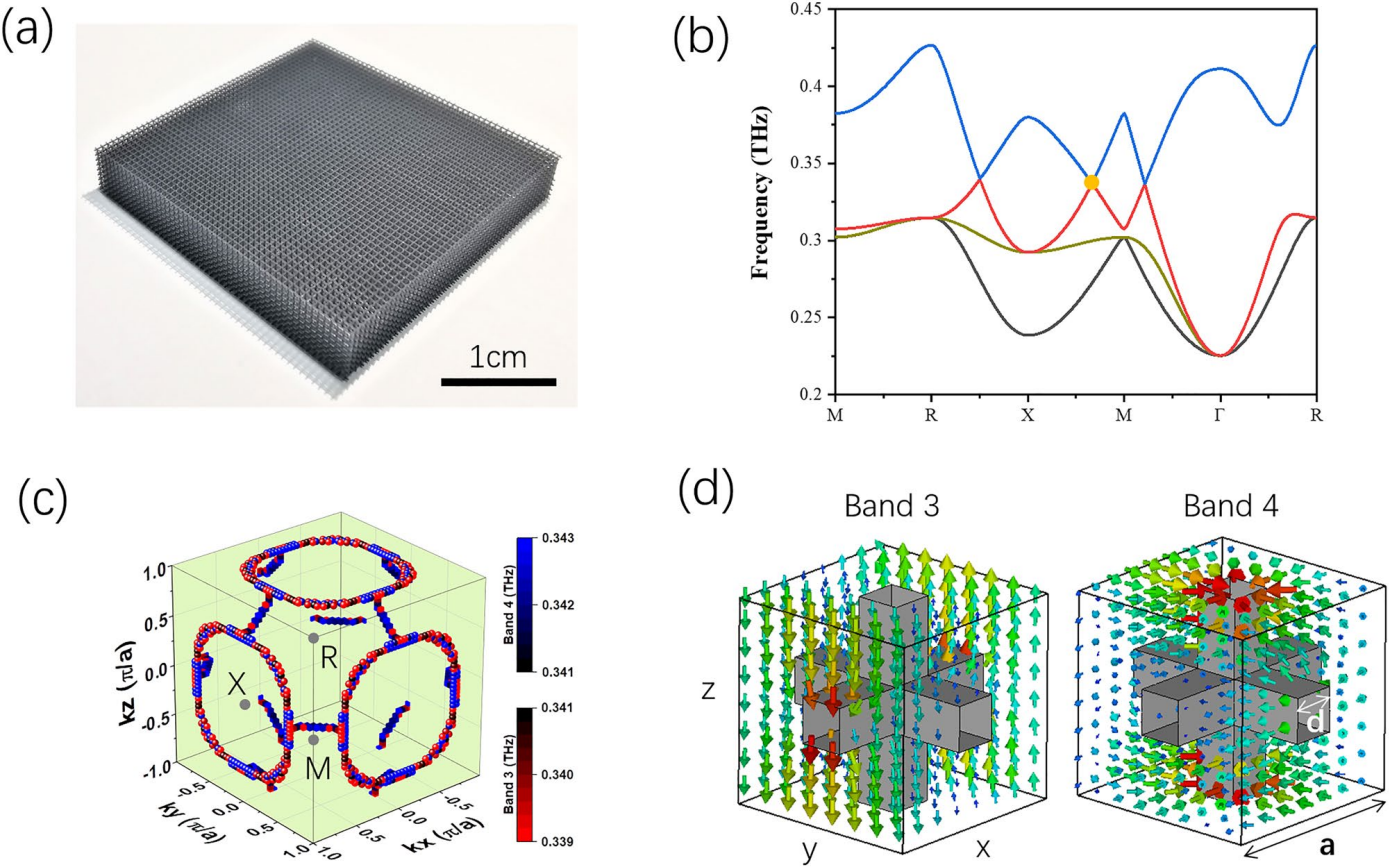
(**a**) Photo of the photonic crystal (before metallization) which is a simple cubic lattice structure with lattice constant, ${\text{a}} = 580\;\upmu {\text{m}}$, and the width of the square rod, ${\text{d}} = 130\;\upmu {\text{m}}$. The picture of the unit cell is shown in (**d**). The sample is about 30 mm × 30 mm in transverse size and about 5 mm (9 unit cells) in thickness. (**b**) Band structure along high symmetric paths in the Brillouin zone, where the 3rd and 4th bands are degenerate at 0.341THz. The positions of high symmetric points in the Brillouin zone are labeled in (**c**). (**c**) Three-dimensional view of band 3 and 4 in the first Brillouin zone around 0.341THz, where the degeneracy presents the nodal chain geometry. (**d**) The arrow plots of the electric field eigenmodes of band 3 and 4 at the degenerate point along XM path, as labeled by orange dot in (**b**).

Our design is a 3D metallic PhC with sub-millimeter feature size, which is fabricated through 3D printing of polymer framework and subsequent chemical plating metallization. Shown in Fig. 1a, the simple cubic lattice structure has the lattice constant, ${\text{a}} = 580\;\upmu {\text{m}}$, and the size of the square rod, ${\text{d}} = 130\;\upmu {\text{m}}$, seeing the unit cell in Fig. 1d. The band structure of the metallic PhC is calculated in a simulation software (CST Microwave Studio), using periodic boundary condition along three dimensions and PEC approximation, and the results are plotted in Fig. 1b,c. In the band structure along high symmetric path in the Brillouin zone, seeing Fig. 1b, we identify three degeneracy points formed by the crossing between band 3 and 4 and being located at 0.341THz. In fact, they belong to a global geometry of NC in the 3D Brillouin zone, seeing Fig. 1c, where three big rings are centered around X points and three small rings around M points considering the periodicity of Brillouin zone^21^. Because of slight frequency dispersion and limited numerical calculating precision, the NC presents a tiny variation in chain diameter, and is not as smooth as mathematical chain model. It is known that the crossing between the 3rd and 4th bands is enforced by mirror symmetry *M*_z_ : {x, y, z} → {x, y, -z}^21^. Therefore, two eigenmodes of the degeneracy along XM path, illustrated in Fig. 1d, show even and odd parities of electric field (E-field) with respect to the z = 0 plane.

**Figure 2:**
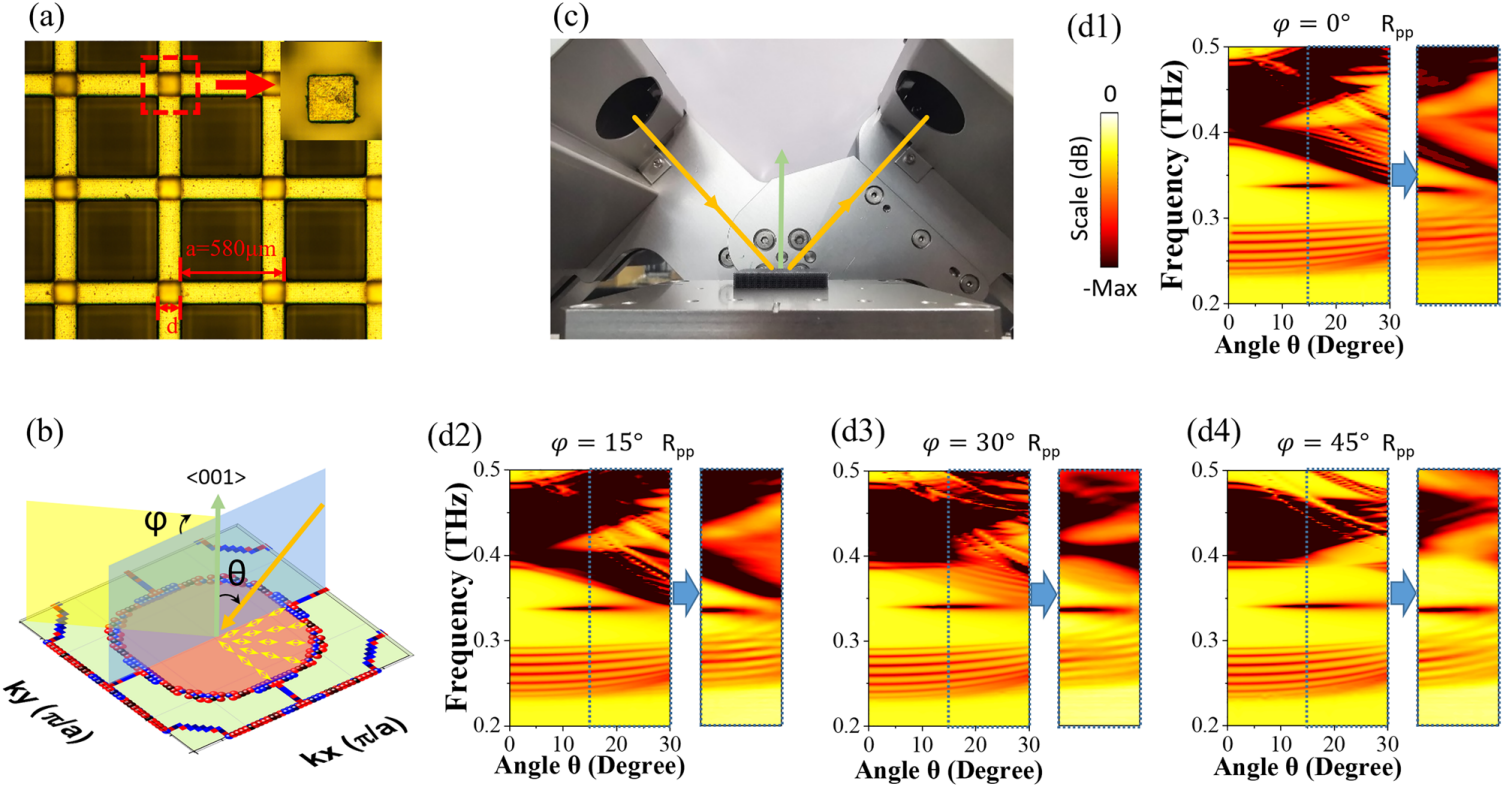
(**a**) Photo of the photonic crystal (after metallization) which is taken along 〈001〉 crystal direction. The inset shows the zoomed cross-section of the square rod on [001] surface. (**b**) Schematic picture of the 〈001〉 projection of the nodal chain (solid symbols, referring to band 3 and 4 in Fig. 1c) and the drumhead surface state (pink area) in k_x_-k_y_ plane, where yellow arrows denote far-field polarization of the drumhead surface state and only one quadrant of polarization texture is illustrated due to C_4_ symmetry. The incident wave vector (orange arrow) and two incident planes (blue and yellow planes) are also depicted for angle-resolved far-field reflection measurement. θ is the incident angle, and φ is the rotating angle of incident plane with respect to 〈001〉 direction. (**c**) Photo of the reflection measurement. (**d1**–**d4**) Measured and simulated far-field reflection, R_pp_, with E-field polarized in the incident plane for both incoming and outgoing waves (p-waves). In each panel, left diagrams with θ ranging from 0° to 30° are simulated results, and right ones with θ ranging from 15° to 30° are measured results. Both comparisons are labeled by dashed regions and blue arrows. For simulation to match well with measurement, the size, d, of the metallic rods has been fine-tuned being 125 μm in numerical calculation.

The sample is made by two steps of processes. First, a simple cubic lattice of photopolymer framework is produced via 3D printing with printing precision ± 2.5 μm. Then, the polymer structure is coated with a thin layer of nickel through chemical nickel plating, i.e., electroless nickel plating. The resultant nickel layer is ~ 3 μm in thickness, far exceeding the skin depth at experimental frequencies, and effectively converts the framework into a metallic PhC. The total size of the whole metallic lattice is about 30 mm × 30 mm × 5 mm, and its top view along 〈001〉 direction is shown in Fig. 2a.

Due to bulk-edge correspondence in topological semimetals, the DSS is formed in the boundary of the lattice^21^. In Fig. 2b, we schematically plot the 〈001〉 projection of the NC and the resultant DSS at the [001] surface. In this work, we are concentrated on the DSS centered at Γ point in surface Brillouin zone, since it is most readily accessible with far-field detection and is free of diffraction beam under small and moderate incident angles of illumination. Two angles are crucial to implement far-field measurement, being the incident angle θ and the rotating angle φ, as depicted in Fig. 2b. The latter defines the orientation of the incident plane with respect to the 〈001〉 crystal direction, while the former gives rise to the DSS-matching momentum k_//_ = k_0_sinθ, where the wave vector in vacuum k_0_ = ω/c with ω being angular frequency and c being the speed of light in vacuum.

In experiment, we used a THz time-domain spectral system (Advantest TAS7400) to measure the reflection spectrum of the [001] surface of the sample. Being a pump-probe detecting system, the spectrometer generates and detects the pulsed THz waveform in time domain, and reflection is obtained from the Fourier-transformed signal recorded from the sample surface. The measuring setup is shown in Fig. 2c where the THz beam from the emitting module is obliquely incident upon the sample and the reflected beam is received by the receiving module. The sample is located at the sample stage, and during measurement we rotate it around the normal direction of the sample surface by the angle φ. When $\tt {{\upvarphi }} = 0^{\circ}$, the incident plane is along the ГX direction. Because of the factory-preset p-wave polarization and the modules’ bulky volume in the spectrometer, only R_pp_ with θ > 15° can be measured, and the measuring results are plotted in Fig. 2d1–d4, where a flat (θ-independent and φ-independent) reflection dip appears at 0.34THz for four values of φ. It is noted that the dip is located in the region between two bulk band projections. The dip is caused by the DSS, and its angle-independence reveals non-dispersion or flatness of the DSS.

**Figure 3:**
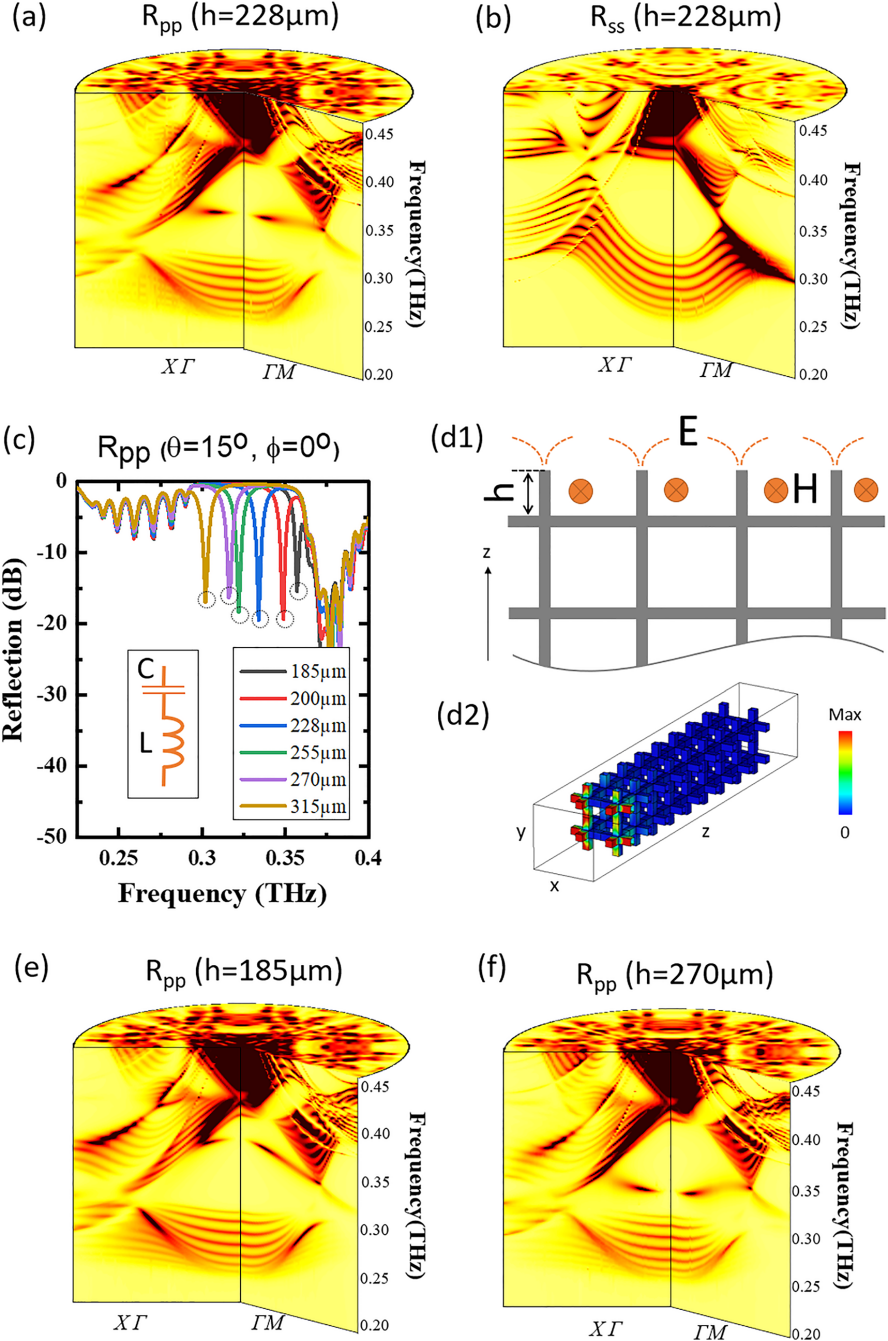
(**a**) and (**b**) Simulated far-field reflection R_pp_ and R_ss_ for both incident planes along ГX (φ = 0°) and ГM (φ = 45°) direction, respectively. (**c**) The effect on reflection of the sectioned metallic rods at the outmost surface cell with varying length, *h*, as depicted in (**d1**). The length, varying from 185 μm to 315 μm, influences the DSS frequency, which can be understood qualitatively by a circuit model shown in the inset. The DSS dips are marked by the dotted circles for different *h* values. (**d1**–**d2**) Schematics of EM fields of the DSS and simulated contour plot of E-field at 0.34THz for R_pp_ with θ = 15°, φ = 0°, and *h* = 228 μm. (**e**) and (**f**) Simulated far-field reflection R_pp_ for *h* = 185 μm and *h* = 270 μm, respectively.

In order to verify the measuring results, we conduct an angle-resolved reflection simulation, where the finite number of unit cells in z direction are modeled and the periodic boundary condition is imposed only in x and y directions. In order to account for the material loss in the metallic PhC and meanwhile minimize the computing time, we set the finite conductivity for nickel through surface impedance parameter. The simulated R_pp_ results are shown in Fig. 2d1–d4, and it is seen that both simulation and experiment are in good agreement after considering a fabrication tolerance of ~ 5 μm. We also simulate R_ss_ where s-wave with E-field perpendicular to the incident plane is incoming and s-wave is outgoing. In sharp contrast to R_pp_, there is no reflecting dip appearing, as shown in Fig. 3b. Furthermore, R_ps_ and R_sp_ are calculated, and both show nearly null feature in the measuring frequencies as both polarizations are orthogonal to each other. Therefore, the sole sensitivity of the DSS dip to p-wave polarization points to a monopole configuration of polarization vectors with monopolar center being Γ point, as illustrated schematically by double arrows in Fig. 2b.

## Analysis and discussion

### Flatness of photonic drumhead surface state

It has been revealed that the dispersion of surface state in electronic systems is sensitive to the surface decoration, even leading to Lifshitz transition of surface states in some topological semimetals^29,30^. Here, the dispersion of the photonic DSS can also be tuned significantly by a simple surface characteristic, as depicted in Fig. 3d1, that is the extruding length, *h*, of the sectioned metallic rod on the surface.

It is seen from both measured and simulated results in Figs. 2 and 3a that the DSS is a flat (zero-dispersive) band being located at ~ 0.34THz. The flatness takes place for the case of *h* = 228 μm. Figure 3c shows the effect of the length on the DSS frequency, where the DSS dip shifts towards lower frequencies when increasing *h*. When *h* is extended above 315 μm, the dip begins to fuse into the first passband lying in ~ 0.22–0.3THz, representing DSS red-shifts into the bulk band. In contrast, when *h* is 185 μm, the dip has blue-shifted into the second passband starting from ~ 0.36THz. Figure 3e,f shows, respectively, a perspective view of simulated band diagrams with negatively dispersive DSS where *h* = 185 μm and positively dispersive DSS where *h* = 270 μm.

Such surface property can be qualitatively explained by a LC circuit model, seeing the inset in Fig. 3c. The capacitance, *C*, comes essentially from the charges accumulated in the end of the rod, and the inductance, *L*, is mainly induced by the current flowing along the sectioned rod, sensitive to the length *h*, seeing the illustration of EM field in Fig. 3d1^31,32^. Therefore, the DSS frequency near Γ point is $\tt \omega_{DSS} = \frac{1}{{\sqrt {LC} }}$. On the other hand, $\tt \omega_{DSS}$ far away from Γ point is pinned to the NC frequency $\tt \omega_{NC}$ which is determined by bulk geometry of the lattice and is robust to *h.* Therefore, *h* can tune $\tt \omega_{DSS}$ being either lower than or equal to or higher than $\tt \omega_{NC}$, giving rise to either positively or zero- or negatively dispersive band. The simple physical picture is supported by the simulation, and Fig. 3d2 shows the simulated contour plot of E-field at 0.34THz for R_pp_ with θ = 15°, φ = 0°, and *h* = 228 μm. The DSS appears clearly on the front [001] surface, upon which the incident wave is impinging, and the field configuration is consistent with the schematic drawing in Fig. 3d1.

### Boundness of photonic drumhead surface state

Another feature of the DSS in simulated and measured results is its vanishing behavior as approaching to Γ point. In other words, it is decoupling from the far field. In fact, the DSS at Γ point is the BIC, where even parity of the mode is incompatible with odd parity of free-space radiative modes, and it belongs to the symmetry-protected BICs. It has been revealed that appearance of BIC is accompanied by a singular point in polarization map^26,27^. In the DSS band, such singularity is seen at Γ point, as illustrated in Fig. 2b, and the monopole configuration of the polarization texture also implies a topological charge of 1.

One technical merit of BIC is extraordinarily high quality (Q-) factor which goes to infinity in ideal case. In order to evaluate the Q-factor, we employ the standard temporal coupled mode theory^4^, where the DSS lying in the bulk bandgap is regarded as single-mode resonator and couples its energy to the radiative mode (p-polarized far field) through reflection,

1 $$\tt r = \frac{{\left( {\frac{1}{{\tau_{r} }} - \frac{1}{{\tau_{nr} }}} \right) + i\left( {\omega - \omega_{DSS} } \right)}}{{\left( {\frac{1}{{\tau_{r} }} + \frac{1}{{\tau_{nr} }}} \right) - i\left( {\omega - \omega_{DSS} } \right)}}$$

Here, *r* denotes reflection coefficient, $\tt i^{2} = - 1$, $\tt \tau_{r}$ and $\tt \tau_{nr}$ characterize energy loss of the resonator due to radiative and non-radiative channel, respectively. $\tt \tau_{r}$, $\tt \tau_{nr}$, and $\tt \omega_{DSS}$ are numerically obtained through fitting the simulated angular reflection spectra by Eq. (1), and then the radiative Q-factor $\tt Q_{r} = \omega_{DSS} \tau_{r} /2$ and the non-radiative Q-factor $\tt Q_{nr} = \omega_{DSS} \tau_{nr} /2$ are calculated.

**Figure 4:**
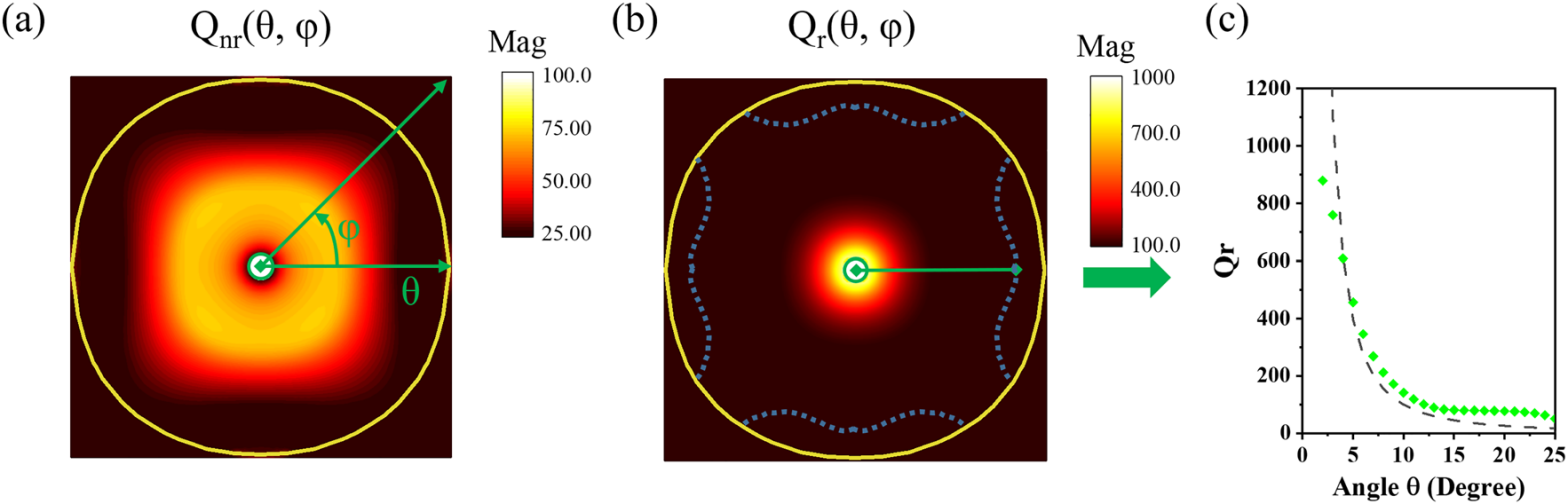
(**a**) Non-radiative Q-factor $\tt Q_{nr}$ map and (**b**) radiative Q-factor $\tt Q_{r}$ map of the DSS at surface Brillouin zone, parametered in polar format with coordinates θ and φ, where Γ point is at the center (green dot) of the map and the radial direction (green arrow) away from the center represents the angle θ increasing from 2° (small green circle) to the angular position of the bulk band projection. For reference, the yellow loops denote the angular position of the NC projection, and the dot lines in (**b**) label the angular positions where the DSS meets the edge of the bulk band. (**c**) $\tt Q_{r}$ versus θ along ГX direction (φ = 0), as denoted by the green line section in (**b**). The symbols are the numerically obtained $\tt Q_{r}$, and the dash line represents the function plot $\tt 1/\sin^{2} \theta$.

Figure 4a,b shows, respectively, the $\tt Q_{r}$ and $\tt Q_{nr}$ map obtained from the reflection around Γ point where the radial direction away from the center of the map represents the angle θ ranging from 2° to the angular position of the NC projection. Note in Fig. 4a,b that the $\tt Q_{r}$ and $\tt Q_{nr}$ data is absent within the small green circles where θ is less than 2° and the DSS dip in R_pp_ becomes too weak to be fitted accurately. It is seen that compared with the low $\tt Q_{nr}$, $\tt Q_{r}$ is relatively high, especially near the center region. In particular, $\tt Q_{r}$ rises rapidly when θ decreases, as plotted in Fig. 4c. Such characteristic of $\tt Q_{r}$ manifests the BIC at Γ point, and can also be understood from aforementioned LC picture. The open LC circuit, i.e., sectioned metallic rod, radiates like a dipole antenna at frequency $\tt \omega_{DSS}$, and thus the [001] surface is just an antenna array with a square lattice of z-orientated dipoles. According to the antenna theory, the far-field radiation pattern of the array becomes that of the individual element when the polar angle θ is zero and near zero, because the array factor is equal to constant^33^. According to the power distribution factor, $\tt \sin^{2} \theta$, of an individual dipole antenna, the far field is null at θ = 0°, and thus the DSS becomes the BIC. A simple comparison between the numerical $\tt Q_{r}$ and the function plot $\tt 1/\sin^{2} \theta$ displays a qualitative agreement, as shown in Fig. 4c.

## Conclusion

In conclusion, we have studies the DSS of the metallic simple cubic lattice at THz frequencies by angle-resolved far-field reflection measurement and numerical simulation. In particular, the DSS persists in the radiation continuum, displays the flatness property in band dispersion, and manifests a symmetry-protected BIC at the center of surface Brillouin zone, which are not identified in previous study. Such properties are of great significance to manipulation of THz wave in planar surfaces and technical applications like THz sensing.

## References

[1] Ashcroft NW, Mermin ND (1976). Solid State Physics.

[2] Castro Neto AH, Guinea F, Peres NMR, Novoselov KS, Geim AK (2009). The electronic properties of graphene. Rev. Mod. Phys..

[3] Armitage N, Mele E, Vishwanath A (2018). Weyl and Dirac semimetals in three-dimensional solids. Rev. Mod. Phys..

[4] Joannopoulos JD, Johnson SG, Winn JN (2011). Photonic Crystals: Molding the Flow of Light.

[5] Cui TJ, Tang WX, Yang XM, Mei ZL, Jiang WX (2016). Metamaterials: Beyond Crystals, Noncrystals, and Quasicrystals.

[6] Lu L, Joannopoulos JD, Soljacic M (2014). Topological photonics. Nat. Photonics.

[7] Lu L, Wang Z, Ye D, Ran L, Fu L, Joannopoulos JD, Soljacic M (2015). Experimental observation of Weyl points. Science.

[8] Chen W-J, Xiao M, Chan CT (2016). Photonic crystals possessing multiple Weyl points and the experimental observation of robust surface states. Nat. Commun..

[9] Yang B, Guo Q, Tremain B, Liu R, Barr LE, Yan Q, Gao W, Liu H, Xiang Y, Chen J, Fang C, Hibbins A, Lu L, Zhang S (2018). Ideal Weyl points and helicoid surface states in artificial photonic crystal structures. Science.

[10] Gao W, Yang B, Tremain B, Liu H, Guo Q, Xia L, Hibbins AP, Zhang S (2018). Experimental observation of photonic nodal line degeneracies in metacrystals. Nat. Commun..

[11] Wang H-X, Chen Y, Hang ZH, Kee H-Y, Jiang J-H (2017). Type-II Dirac photons. NPJ Quantum Mater..

[12] Hu C, Li Z, Tong R, Wu X, Xia Z, Wang L, Li S, Huang YZ, Wang S, Hou B, Chan CT, Wen W (2018). Type-II Dirac photons at metasurfaces. Phys. Rev. Lett..

[13] Yang Y, Gao Z, Feng X, Huang Y-X, Zhou P, Yang SA, Chong Y, Zhang B (2020). Ideal unconventional Weyl point in a chiral photonic metamaterial. Phys. Rev. Lett..

[14] Pendry JB, Aubry A, Smith DR, Maier SA (2012). Transformation optics and subwavelength control of light. Science.

[15] Chen H-T, Taylor AJ, Yu N (2016). A review of metasurfaces: physics and applications. Rep. Prog. Phys..

[16] Glybovski SB, Tretyakov SA, Belov PA, Kivshar YS, Simovski CR (2016). Metasurfaces: from microwaves to visible. Phys. Rep..

[17] Birks TA, Knight JC, Russell PSJ (1997). Endlessly single-mode photonic crystal fiber. Opt. Lett..

[18] Lu L, Gao H, Wang Z (2018). Topological one-way fiber of second Chern number. Nat. Commun..

[19] Fang C, Weng H, Dai X, Fang Z (2016). Topological nodal line semimetals. Chin. Phys. B..

[20] Wang S, Wu W, Yang S (2019). Progress on topological nodal line and nodal surface. Acta Phys. Sin.-Ch. Ed..

[21] Yan Q, Liu R, Yan Z, Liu B, Chen H, Wang Z, Lu L (2018). Experimental discovery of nodal chains. Nat. Phys..

[22] Hsu CW, Zhen B, Stone AD, Joannopoulos JD, Soljacic M (2016). Bound states in the continuum. Nat. Rev. Mater..

[23] Lee J, Zhen B, Chua S, Qiu W, Joannopoulos JD, Soljacic M, Shapira O (2012). Observation and differentiation of unique high-Q optical resonances near zero wave vector in macroscopic photonic crystal slabs. Phys. Rev. Lett..

[24] Hsu CW, Zhen B, Lee J, Chua S, Johnson SG, Joannopoulos JD, Soljacic M (2013). Observation of trapped light within the radiation continuum. Nature.

[25] Hsu CW, Zhen B, Chua S, Johnson SG, Joannopoulos JD, Soljacic M (2013). Bloch surface eigenstates within the radiation continuum. Light-Sci. Appl..

[26] Zhen B, Hsu CW, Lu L, Stone AD, Soljacic M (2014). Topological nature of optical bound states in the continuum. Phys. Rev. Lett..

[27] Zhang Y, Chen A, Liu W, Hsu CW, Wang B, Guan F, Liu X, Shi L, Lu L, Zi J (2018). Observation of polarization vortices in momentum space. Phys. Rev. Lett..

[28] Li Z, Wu J, Huang X, Lu J, Li F, Deng W, Liu Z (2020). Bound state in the continuum in topological inductor–capacitor circuit. Appl. Phys. Lett..

[29] Yang HF, Yang LX, Liu ZK, Sun Y, Chen C, Peng H, Schmidt M, Prabhakaran D, Bernevig BA, Felser C, Yan BH, Chen YL (2019). Topological Lifshitz transitions and Fermi arc manipulation in Weyl semimetal NbAs. Nat. Commun..

[30] Ekahana SA, Li YW, Sun Y, Namiki H, Yang HF, Jiang J, Yang LX, Shi WJ, Zhang CF, Pei D, Chen C, Sasagawa T, Felser C, Yan BH, Liu ZK, Chen YL (2020). Topological Lifshitz transition of the intersurface Fermi-arc loop in NbIrTe4. Phys. Rev. B.

[31] Pendry JB, Holden AJ, Stewart WJ, Youngs I (1996). Extremely low frequency plasmons in metallic mesostructures. Phys. Rev. Lett..

[32] Pendry JB, Holden AJ, Robbins DJ, Stewart WJ (1999). Magnetism from conductors and enhanced nonlinear phenomena. IEEE Trans. Microwave Theory Tech..

[33] Balanis CA (2005). Antenna Theory.

